# Cautery Disbudding Iron Application Time and Brain Injury in Goat Kids: A Pilot Study

**DOI:** 10.3389/fvets.2020.568750

**Published:** 2021-01-18

**Authors:** Melissa N. Hempstead, Jan K. Shearer, Mhairi A. Sutherland, Jennifer L. Fowler, Joseph S. Smith, Jodi D. Smith, Taylor M. Lindquist, Paul J. Plummer

**Affiliations:** ^1^Veterinary Diagnostic and Production Animal Medicine, College of Veterinary Medicine, Iowa State University, Ames, IA, United States; ^2^Animal Behaviour and Welfare, AgResearch Ltd., Ruakura Research Centre, Hamilton, New Zealand; ^3^Veterinary Clinical Sciences, College of Veterinary Medicine, Iowa State University, Ames, IA, United States; ^4^Idexx Laboratories, Westbrook, ME, United States; ^5^Biomedical Sciences, College of Veterinary Medicine, Iowa State University, Ames, IA, United States; ^6^Veterinary Pathology, College of Veterinary Medicine, Iowa State University, Ames, IA, United States

**Keywords:** magnetic resonance imaging, histopathology, welfare, caprine, small ruminant, goat, disbudding

## Abstract

Cautery disbudding is a painful procedure performed on goat kids to prevent horn growth that may result in brain injury. Thermal damage to the cerebral cortex of the brain and subsequent neurologic disease is a primary concern. Cautery iron application time may affect transmission of heat to the brain; however, research in this area is scarce. Therefore, the objective of this pilot study was to evaluate the effect of iron application time on brain injury of goat kids. A total of six buck and doe kids <9 days of age were obtained from a commercial dairy and transported to an Iowa State University research facility. Kids received a different randomly assigned application time (5, 10, 15, or 20s) on each horn bud. Kids were disbudded using an electric cautery iron (under isoflurane general anesthesia). After a 5-day observation period, the kids were euthanized, and magnetic resonance (MR) images were acquired to evaluate brain injury. Additionally, four of the six kids were presented for gross examination and two kids were selected for histopathologic examination. From the MR images, white matter edema was observed subjacent to four treated areas, representing application times of 5 s (one horn bud), 15 s (one horn bud), and 20 s (two horn buds). With the exception of the horn bud that received 5 s, which had white matter edema restricted to a single gyrus, the remaining three groups had a branching region of edema. No bone abnormalities were identified on any kids. Gross evidence of discoloration and hemorrhage on the cerebral hemispheres was observed on two horn buds that received 20 s, two horn buds that received 15 s, and one horn bud that received 10 s. Microscopic lesions consisting of leptomeningeal and cerebrocortical necrosis were observed in sections of brain from all groups. Lesions were most severe with 20 s. In conclusion, all application times used in this study resulted in some level of brain injury; however, using 15 s or more resulted in more severe and consistent brain injury. These results indicate that extended iron application time may increase the risk of brain injury in cautery disbudded kids.

## Introduction

Cautery disbudding is routinely performed on dairy goat kids to destroy the horn buds and prevent horn growth. Horns can increase the risk of injuries to other goats during agonistic encounters or stock persons during handling (1), and the amount of space required at the feed rack (2). Although it is possible to manage horned goats by adapting facilities and management practices, until practice change is adopted by farmers, disbudding (with pain relief) remains the best option [see review by (3)]. It is generally accepted that cautery disbudding causes pain, and reduces goat welfare (4–6). Goat kids perform increased leg shaking and vocalization frequencies during cautery disbudding (7, 8) and show substantial increases in cortisol concentrations following the procedure (5, 9). Tissues surrounding the horn bud have increased sensitivity to pressure for up to 1 h after disbudding (10). Therefore, effective pain management should be included as part of routine procedure as is mandated for calves in many countries. In addition to the use of pain mitigation strategies, cautery disbudding practices should be optimized in order to reduce any other potential detrimental effects associated with cautery disbudding.

Cautery disbudding can cause thermal damage, leading to bacterial meningoencephalitis, and necrosis of the cerebrum in goat kids. These effects can manifest within days (11, 12), weeks (13–15) or even up to 2 months after the procedure (16). Injuries that are commonly associated with improper cautery disbudding practice include lesions of the cerebral cortex underlying the horn bud and frontal bone, necrosis, thrombosis and suppurative inflammation (11–13, 16, 17). There are reports of a number of goat kid mortalities associated with cautery disbudding-related injuries: in one study, 12/150 kids (8%) died from injuries associated with cautery disbudding within 3 days (12). In another study, that monitored 1,262 kids from 16 farms, of the kids that died between birth and 1 year of age, 15.9% (17/107; 1.3% of kids enrolled) died as a result of cautery disbudding-related injuries within 12 weeks (14). Although a high level of training and experienced personnel can generally minimize mortality, it is imperative that more scientifically validated information, such as suitable iron application time, is provided to cautery disbudding operators to ensure that their practice does not increase the risk of causing brain injury.

Alternatives to cautery disbudding that do not result in thermal meningoencephalitis do exist, such as caustic paste and cryosurgical disbudding (18, 19); however, they cause more pain and are less efficacious in preventing horn regrowth or scurs (20). Scurs are the result of incomplete destruction of the horn bud and characterized by partial regrowth of horn tissue that are not usually attached to the underlying skull (21). Therefore, the most viable option to date for preventing horn growth in kids, is cautery disbudding.

To the authors' knowledge, there is no scientific literature available on the effect of cautery iron application time on thermal injury caused to the brain of goat kids. Therefore, the objective of this pilot study was to evaluate the effect of cautery iron application time on brain injury of goat kids. Application times ranging from 5 to 20 s were evaluated, based on application times reported in previous studies on disbudded goat kids [see review by (3)]. Our a priori hypothesis was that 15 and 20 s application times will result in more substantial brain injury and that 5 and 10 s application times will not result in brain injury.

## Materials and Methods

This pilot study was approved by the Institutional Animal Care and Use Committee at Iowa State University prior to data collection (Protocol number: IACUC-19-193). If an animal displayed clinical signs of neurological injury (e.g., convulsions, altered mentation, or blindness) prior to the 5-day observation period, they were euthanized; this was not required.

### Animals and Housing

Six Saanen and Saanen cross goat kids of either sex (three males; three females) were used in this study. They were aged between 6 and 9 days old and had a mean weight of 4.4 kg (SE: 0.3 kg; 3.3–5.3 kg). The animals were acquired between June and August 2019 (in groups of 2) from a commercial farm in Wisconsin and transported to the Laboratory Animal Resources facility of Iowa State University in Ames, Iowa, where the study was conducted. Both sexes were used as these animals were available at the time of the study and chosen by the farm. Non-steroidal anti-inflammatory drugs were not provided for the kids during disbudding, as this may have affected the results (i.e., inflammation). Additionally, local anesthesia (e.g., ring or nerve blocks) were not carried out due to apparent inefficacy and resultant stress caused in goat kids (4, 9, 22).

The kids were raised in pairs and bottle-fed a total of 300–440 mL milk replacer (custom-milled milk replacer supplied by the producer) 2–3 times daily. Water was available *ad libitum* in a plastic container and the pen was bedded with straw, which was refreshed between pairs of kids; no roughage was provided.

After a 24-h habituation period, the kids were cautery disbudded. Disbudding was carried out at 8:00 a.m. each treatment day (described below). The average temperature and humidity at disbudding was 24.3 ± 0.2°C (mean ± SD; range: 24.7–24.1°C) and 67.7 ± 6.2% rh (mean ± SD; range: 78.8–58.6% rh). After disbudding, the kids were returned to their pen and stayed with the same pen-mate for 5 days, at which point they were euthanized for assessment of skull and brain injury (discussed below).

### Experimental Design

Disbudding was carried out on 3 days (inconsecutive) over a 6-week period, with two kids disbudded per experimental day. At least a day prior to disbudding, kids were assigned an identification number (1–6), which had a predetermined random combination of iron application times (5, 10, 15, or 20 s); two different application times were applied to each kid (Supplementary Table 1). Sex and age were balanced across treatments. The application times were randomized so each combination was represented per day and a different application time was used per horn bud (i.e., two different application times per kid). The left horn bud was always disbudded first. A power analysis was not conducted as this was a pilot study and the intention was to first establish whether brain injury associated with the iron application times used in this study could be detected using MR imaging and histopathology. Additionally, due to the limited number of replicates, statistical analysis was not performed; therefore, descriptive data will be presented.

Hair within the horn bud area was removed using an electric clipper (Oster Professional Care A5 Turbo 2-Speed, Mcminnville, TN) prior to disbudding to improve visibility of the horn bud and to reduce the burning of hair. General anesthesia was induced using isoflurane (via a mask placed over the muzzle) at a rate of 5% until the kid lost consciousness. Unconsciousness was assessed via cornea and palpebral reflexes and confirmed when both were absent. Once unconscious, the kid was maintained for the duration of disbudding at a rate of 2.5% isoflurane with an oxygen flow rate of 3 L/min. After disbudding, the isoflurane supply was removed, allowing inhalation of pure oxygen for <10 s. The face mask was then removed, and the kid remained under veterinary supervision until consciousness was regained (<5 min).

The same experienced operator (MNH) disbudded the kids using an electric disbudding iron (Rhinehart X50, Spencerville, IN) with a 19 mm tip (outer diameter; 13 mm: inner diameter) plugged directly into an electric outlet. The iron used is typical of those used in the United States for disbudding dairy goat kids. The temperature of the iron was not measured, but the iron was allowed to heat for 20 min prior to use. Manufacturer instructions state a range in temperature of 510–567°C. The iron was applied to the horn bud at an angle perpendicular to the skull. Downwards pressure was applied, and a twisting motion utilized until the tip of the iron rested against the skull. A technician counted the seconds aloud so that the operator could remove the horn bud with a flick (as per 5) at the conclusion of iron application. Each animal was placed in lateral recumbency and the head was rotated so that each horn bud was disbudded with the operator's dominant hand (right-handed). The tip was cleaned between horn buds using a wire brush to ensure horn tissue was removed. The iron was allowed a “rest” period (≤10 s) to return to maximal temperature between horn buds. An antibacterial agent was not applied to the horn bud wounds to avoid affecting results.

Kids were monitored for 0.5 h following disbudding to ensure no kids presented neurological signs of distress associated with disbudding (e.g., convulsions, altered mentation, or blindness).

The kids were raised for 5 days following disbudding and the kids' health status (e.g., lethargy, ability to feed, and locomotion) was monitored daily by study personnel and the study veterinarian. The 5-day observation period was deemed appropriate by our group to detect changes in brain tissue consistent with thermal injury associated with iron application. After the 5-day observation period, kids were euthanized with an intravenous dose of sodium pentobarbital (≥18 mg/kg) immediately prior (within 10 min) to magnetic resonance (MR) imaging to avoid development of artifacts that would be seen in the images post-mortem. Death was confirmed via absence of a cornea reflex, as well as cessation of heartbeat and regular respiration.

### Magnetic Resonance Imaging

Multiplanar magnetic resonance images were acquired with a 1.5 T unit (GE Signa, GE Healthcare, Chicago, IL). This required the removal of metal ear tags prior to introducing the cadaver to the imaging suite. Each cadaver was imaged individually. For this, the cadaver was placed in sternal recumbency with the cranium placed within a head coil. Multiplanar images were acquired using a standard protocol including T2-weighting (T2w), T1-weighting (T1w), fluid attenuated inversion recovery (FLAIR), short tau inversion recovery (STIR), and gradient echo (GRE) sequences. Play-Doh® (Hasbro Inc., Pawtucket, RI) was conformed to the lateral aspects of the neck to support straight positioning of the anatomy of interest and to decrease the impact of air gap between the animal and the coil on image quality.

All images acquired were stored on a picture archiving and communication system for assessment by a board-certified veterinary radiologist (JLF) after all cadaver imaging was completed. JLF was blind to the treatment each kid received. Image assessment was performed on a medical grade workstation using E-Film software (E-Film v 4.2.2.1; Merge Healthcare of IBM Watson Health, Cambridge, MA). Images were evaluated for (1) the presence or absence of gray and/or white matter edema (i.e., hyperintensity on T2w persisting on FLAIR), (2) the location of the hyperintensity (if identified), (3) the number of gyri affected, and (4) the shape of the intensity (i.e., round, triangular, or branching) (23). JLF also assessed the presence or absence of transtentorial and foramen magnum herniation, dilations and asymmetries of the ventricular system, focal subarachnoid accumulations of cerebrospinal fluid adjacent to parenchymal abnormalities, and bone abnormalities (e.g., loss of margin distinction, undulant contour, altered patterns of intensity of the cortical bone).

### Gross Examination

Gross examination of four of the kids was performed by a large-animal internal medicine specialist (JSS) following MR imaging, but before histopathological examination. The first two kids were not grossly examined due to logistical challenges. Based on a similar methodology used by Hempstead et al. (10), the horn bud sites were grossly examined to assess exterior tissue damage to the skin and skull (e.g., ulceration, necrosis, and hemorrhage). The skin was then removed, and the skullcap cut away so that both cerebral hemispheres could be exposed. The dorsal surfaces of the cerebral hemispheres beneath the horn bud sites were grossly examined for evidence of injury (e.g., discoloration, indentation) or inflammation.

### Histopathology

Two kids that exhibited evidence of injury to the brain based on gross examination (i.e., discoloration on the brain immediately underneath the disbudding region), were selected for histopathological examination. One horn bud from each iron application time was represented. The anterior part of the brain containing the frontal lobes was collected and fixed in 10% neutral buffered formalin. Histology was carried out by a board-certified pathologist (JDS) from the Iowa State University, College of Veterinary Medicine Pathology Laboratory. JDS was blind to the treatment each kid received. A 5-mm thick section from the affected portion of cerebrum from each brain (i.e., from beneath the horn bud sites) was processed by routine histologic methods, embedded in paraffin, sectioned at 5 μm, and stained with hematoxylin and eosin (H&E), and examined for histopathological changes by light microscopy.

## Results

### Magnetic Resonance Imaging

**Figure 1:**
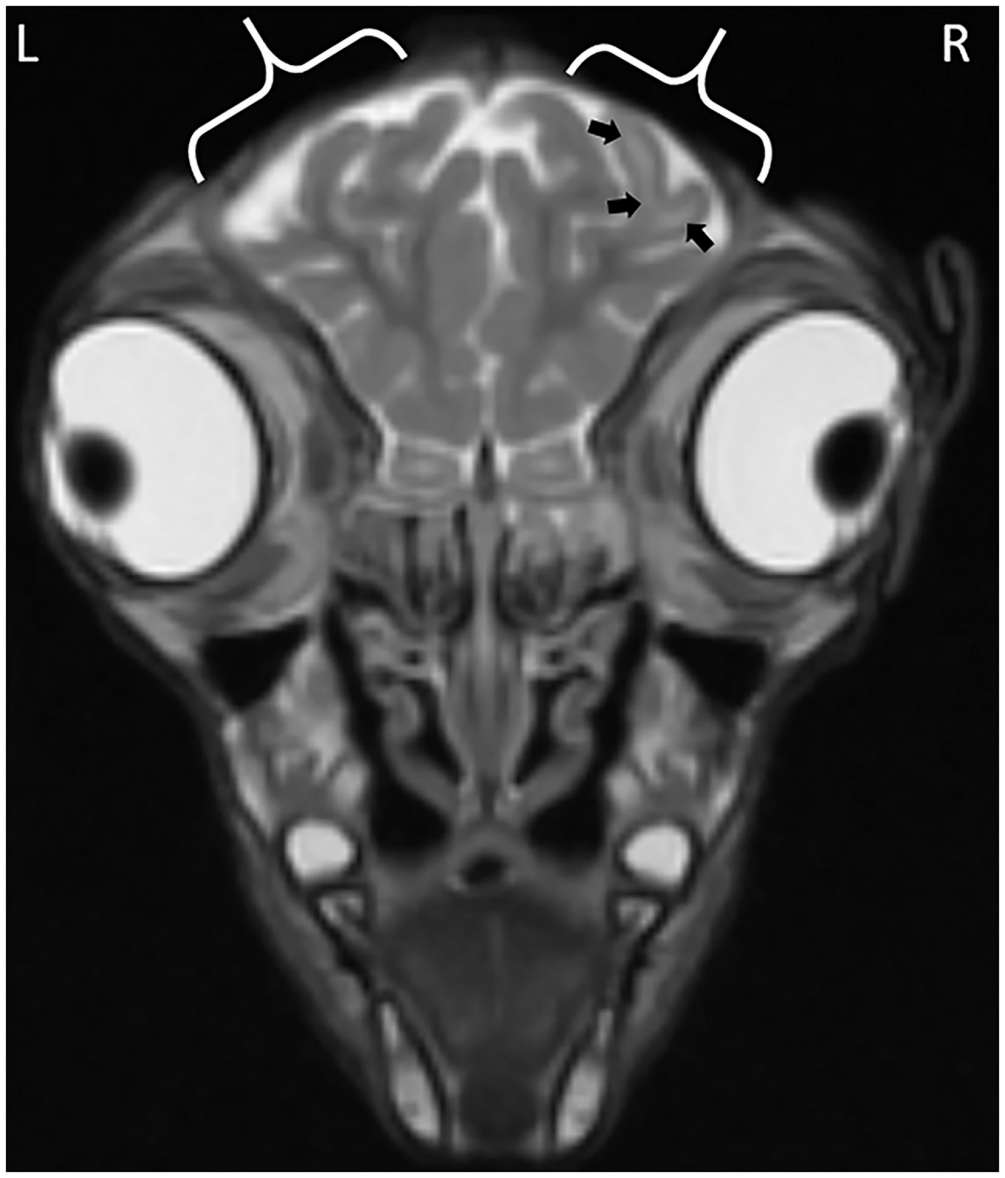
Focal cerebral abnormalities subjacent to the right horn bud that received iron application time of 20 s. The black arrows highlight gyral edema evidenced by the hyperintense (whiter) regions in the white matter of the gyrus. Calipers demark the region of skin removed at disbudding site evidenced by a gap in the midintense skin covering the skull.

**Figure 2:**
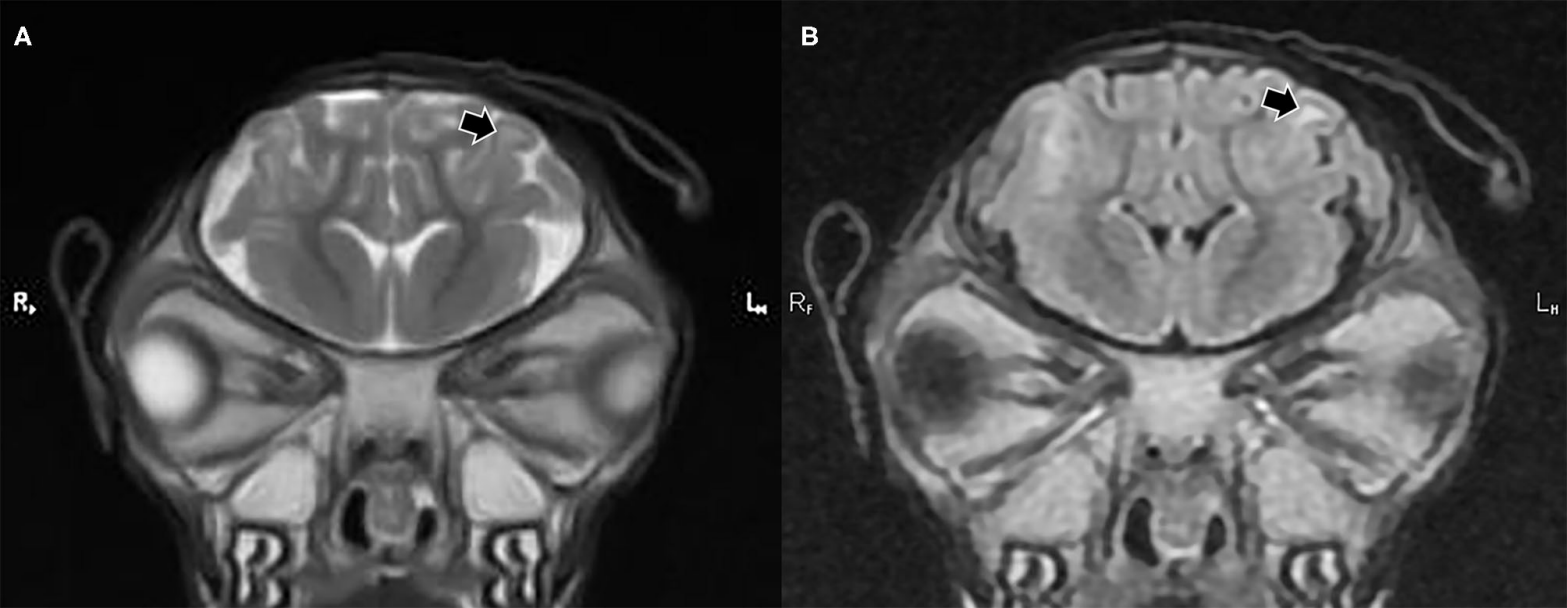
T2 weighted **(A)** and fluid attenuated inversion recovery [FLAIR] transverse plane **(B)** images exhibiting focal cerebral abnormalities subjacent to the left horn bud that received iron application time of 5 s. The black arrows highlight gyral edema evidenced by a hyperintense (whiter) region in the white matter of the gyrus, which remains hyperintense in the FLAIR image.

The observations from the MR images are presented in Supplementary Table 1. For all application time groups, focal regions of superficial skin loss were identified at the site of disbudding. This appeared as a markedly hypointense gap in the midintense soft tissues overlying the skull (Figures 1, 2). The intensity of the gray matter of the frontal lobes remained normal when compared to gray matter of other regions for all application time groups. White matter hyperintensity was observed underlying four horn buds with cautery application times of 5 s (one horn bud), 15 s (one horn bud), and 20 s (two horn buds; Figure 1). The presence T2 hyperintensity that persists on FLAIR is consistent with edema. Beneath one horn bud that received a 5 s application, the region of edema was seen within one gyrus (Figure 2). This is contrasted by a branching region of edema, suggesting more severe edema beneath the other three horn buds treated with a longer application time. No GRE signal voids were observed in the braincase, which suggests that no hemorrhage >12–24-h old was present in the braincase. There were no abnormalities detected from the MR images for three kids treated with 5 s (one horn bud), 10 s (two horn buds), 15 s (two horn buds), and 20 s (one horn bud).

For all kids, the posterior margin of the occipital lobe and cerebellum were normally positioned consistent with an absence of transtentorial and foramen magnum herniation, respectively. No dilations or asymmetries were seen in the ventricular system. Subjectively, the degree of filling of the subarachnoid space with cerebrospinal fluid was uniformly normal in all kids. In some of the sequences a normal signal void (i.e., susceptibility artifact) was seen overlying the frontal bone at the location of the skin necrosis from the disbudding procedure. Where the bone was not obscured by signal voids secondary to skin loss at the location of disbudding, the bone maintained a normal multilayered pattern with a smooth and well-defined margin. The intensity of the cortical bone and diploe at the site of disbudding was identical to the bone in locations distant from the disbudding. As such, no bone abnormalities were identified in any kid.

### Gross Examination

**Figure 3:**
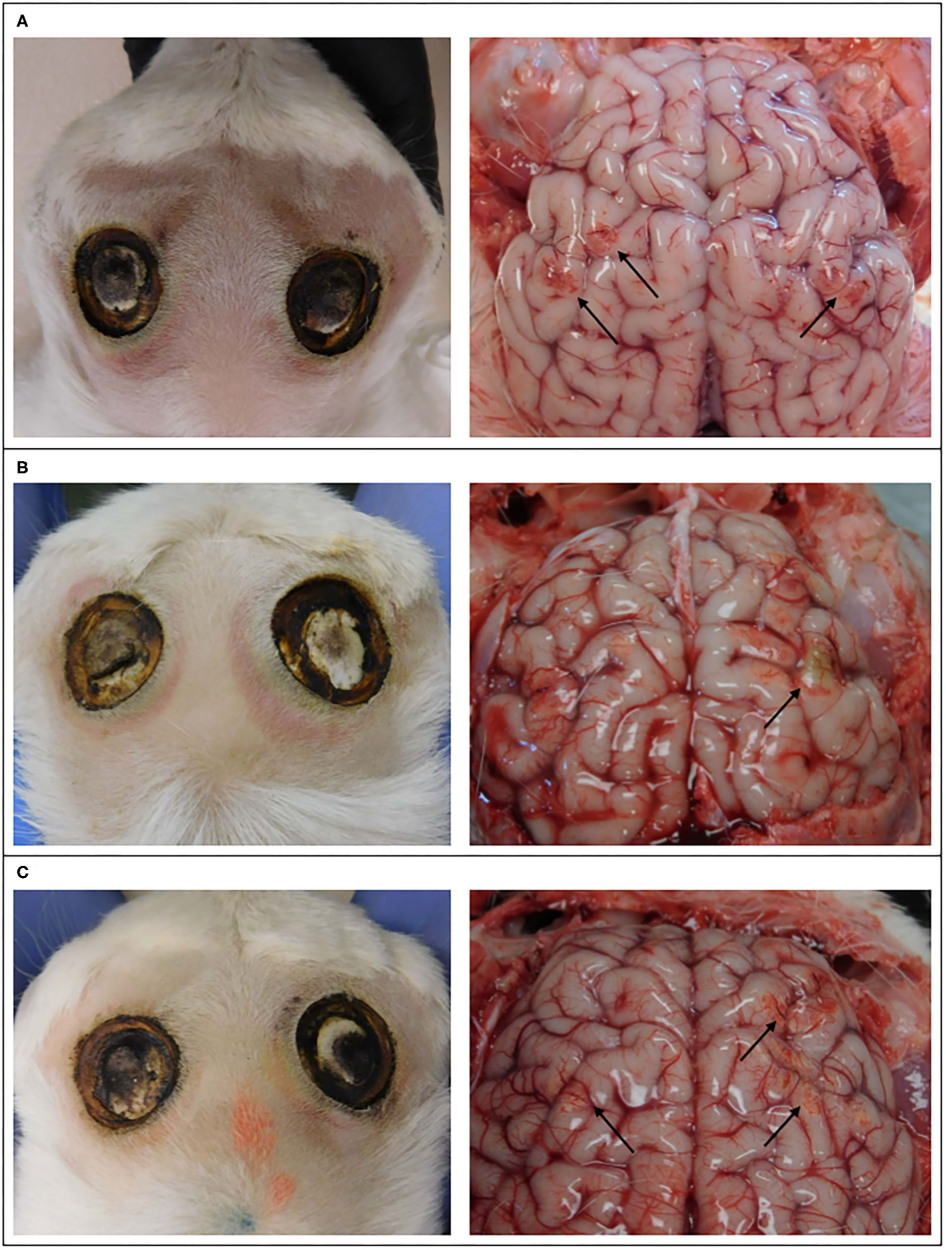
Images of the external surface of the head of goat kids and cerebral hemispheres 4 days after cautery disbudding. These kids received 20 s on left horn bud and 15 s on the right horn bud **(A)**, 5 s on the left horn bud and 20 s on the right horn bud **(B)**, and 10 s on the left horn bud and 15 s on the right horn bud **(C)**. Arrows indicate discoloration and hemorrhage.

Evidence of discoloration and hemorrhage on the cerebral hemispheres was observed subjacent to (1) two horn buds that were treated with 20 s (Figure 3), (2) two horn buds that were treated with 15 s (Figure 3), and (3) one horn bud treated with 10 s (Figure 3). One kid treated with 10 s and 5 s, showed no evidence of gross changes.

### Histopathology

**Figure 4:**
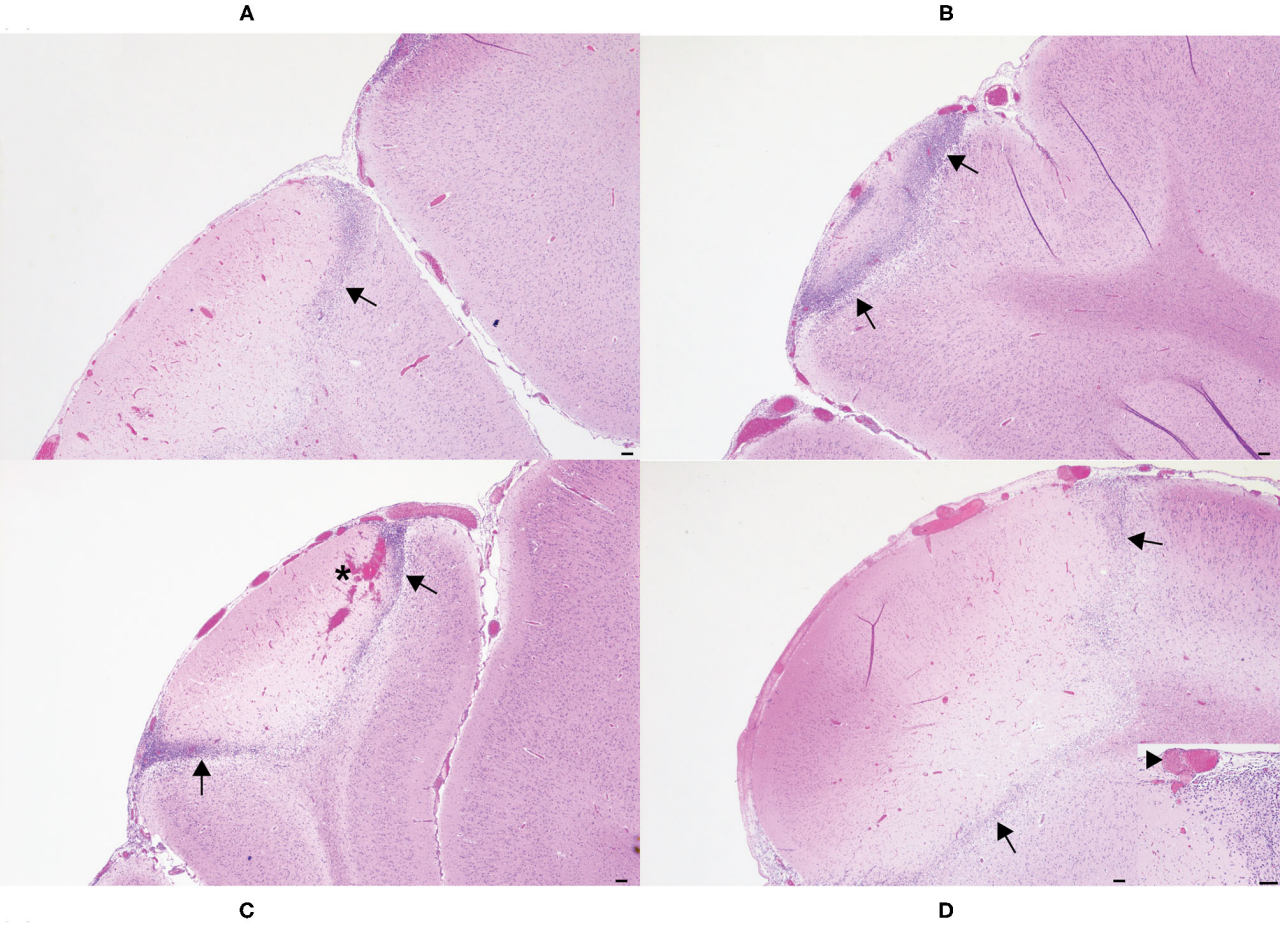
Histologic images of affected cerebral gyri from the 5 s **(A)**, 10 s **(B)**, 15 s **(C)**, and 20 s **(D)** applications. In all application times, the cerebrum subjacent to the horn bud contained areas of leptomeningeal and cortical necrosis, often rimmed by moderate to large numbers of gitter cells (arrows). Hemorrhage was also present in the 15 s application (**C**, asterisk). Occlusive thrombi were identified within leptomeningeal blood vessels of most applications, but most frequently in the 20 s application (**D**, inset, arrowhead). Hematoxylin and eosin. Scale bars = 100 microns.

One H&E-stained section of cerebrum subjacent to each horn bud was examined microscopically. Included in the section was cerebral cortex and underlying white matter. Lesion character was similar in all sections and consisted of necrosis of the leptomeninges and underlying cerebral cortex (Figure 4). Moderate numbers of gitter cells were present at the periphery of necrotic foci, and adjacent viable parenchyma was mildly edematous with reactive changes (gliosis, hypertrophied endothelium). The brain lesion induced by the 20 s application was subjectively more severe than the other application times. Necrosis in the examined section from this horn bud extended through the thickness of the cerebral cortex and into underlying white matter. Leptomeningeal blood vessels also frequently contained occlusive fibrin thrombi. Brain lesions were less extensive in the other application times, but leptomeningeal thrombi were also observed in the 15 and 5 s applications, along with mild hemorrhage in the 15 s application. Lesions were most superficial in the 10 s application, and thrombosis was not observed in this section.

## Discussion

The objective of this pilot study was to evaluate the effect of cautery iron application time on brain injury of goat kids. Although previous literature reported clinical neurological, gross, and histopathological abnormalities in the brain of goat kids following cautery disbudding (11–13, 16), imaging studies to evaluate detectible abnormalities of the brain parenchyma are limited. Therefore, this lack of data limited the feasibility of large prospective studies to evaluate lesion severity and the associated clinical implications, justifying the benefit of pilot studies to provide this information. In line with our hypothesis, goat kids that experienced a longer period of cautery iron placement on the horn bud (15 and 20 s) experienced severe brain injury, which may have resulted from direct thermal injury of the brain parenchyma, or infarction secondary to leptomeningeal thrombosis. Interestingly, there was some evidence that iron application times of 5 and 10 s caused mild to moderate injury based on white matter edema and histopathological changes in the cerebral cortex. The results from our pilot study indicate that extended application times can increase the risk of brain injury. Additionally, further studies are required, with greater sample size to ascertain whether a 5 s application consistently results in brain injury.

Previous literature has documented skull and brain injury consistent with meningoencephalitis associated with cautery disbudding in goat kids (10–17, 20), but these studies did not evaluate the effect that iron application time had on resultant injury. Similar to the findings of the present study, others have reported that there were lesions, hemorrhage, necrosis, and yellowish areas on the cerebral cortex beneath the disbudding sites in five kids that were between 7 and 30 days of age (that died up to 3 weeks after disbudding; 13), 5/12 4- to 10-day-old kids (that were examined post-mortem after disbudding; 12) and three 2-month-old kids (of 139 goats identified with brain lesions; 16). Additionally, the presence of large numbers of inflammatory cells including mononuclear cells and neutrophils as well as fibrin deposits have been reported (11–13).

Previously, goat kids up to 3 weeks following cautery disbudding were reported to exhibit signs of central nervous system depression including incoordination, paraplegia, convulsions, and coma (11, 12). We did not observe any of these signs within 5 days after cautery disbudding. The difference between studies is likely associated with the amount of time animals were observed in the current study, which was likely not long enough for these symptoms to occur. Additionally, we did not find any structural damage to the skull associated with the cautery iron, whereas others have reported this level of damage (11, 13). Other factors may affect the level of skull or brain injury and resultant meningoencephalitis. In the studies discussed, the amount of pressure applied to the head during disbudding may have been more than what was applied in the current study, and may have impacted on the level of skull damage reported (11, 13). Some disbudding irons have a sharp tip and may be more likely to damage or cut through the skull in comparison with the blunt tip that was used in this study (especially if a twisting motion is utilized); however, additional research would be required to determine whether this is true. In the present study, the same operator performed all disbudding, but the amount of pressure applied may have varied between applications. As much as was possible, the operator used a similar amount of force between iron applications, although this was not measured. Future studies should endeavor to standardize the amount of pressure applied, as this may impact on the level of brain injury caused.

Best practice cautery disbudding for dairy calves and goat kids appears to be similar (24, 25); however, calf and kid anatomy is clearly different. The skull bones of goat kids are much thinner than that of calves, which appears to result in reports of thermal injury and meningoencephalitis, whereas for calves, no such reports are known. The brain is much closer to the source of heat and may be more susceptible to thermal injury (13). Cautery disbudding operators that are accustomed to disbudding calves need to exercise caution when disbudding goat kids. It is vital that proper training on how to safely and effectively disbud goat kids is received, and that operators have adequate practice (e.g., on cadavers) prior to disbudding live goat kids. Recommendations from a recent article suggested cautery iron usage should be applied for no more than 5 s (15). In the present study, one kid from the 5 s application showed evidence of white matter edema in MR images. This suggests that some portion of goat kids may experience mild brain edema even if strict adherence to this timing is followed; however, the long-term clinical significance (if any) of this type of injury is unknown at this time. Skull thickness was not obtained during post-mortem assessment. Additionally, skull thickness could not be accurately provided from the MR images due to normal susceptibility artifact occurring secondary to the interface between the air and the skull bone lacking skin coverage due to loss from the disbudding procedure. Susceptibility artifact is a normal phenomenon identified on MR images at the interface between air and bone with a substantially different molecular make-up (26). Susceptibility artifact at this interface impedes identification of the bone margin and therefore precludes accurate measurement in locations where the skin is absent. As a consequence, it remains unclear if the edema seen in one 5 s application is secondary to that kid having a thinner skull or simply a spurious outcome. However, the overall results of the present study suggest that adhering to 5 s or less for cautery application may be largely protective to the adjacent brain tissue. In future studies, skull thickness should be evaluated to provide a greater understanding of the effects of cautery iron damage to all affected tissues.

In all cases, the posterior margin of the occipital lobe and cerebellum were normally positioned consistent with an absence of transtentorial and foramen magnum herniation, respectively. No dilations or asymmetries were seen in the ventricular system. These findings suggest that no kids developed an increased intracranial pressure secondary to the cautery procedure.

The present study used a variety of forms of assessment of brain injury (e.g., MR imaging, gross and histological examination). However, in 4/12 horn buds (~33%), there were differences in the presence or absence of brain injury between MR images and gross and histological assessment (Supplementary Table 2). It is important to note that neuroimaging is adjunctive, and a lack of positive evidence of brain injury does not mean that it is absent (27). A small lesion was observed (from MR images) in the brain of a 2-month-old goat kid that was presented for examination based on depressed mental status and neurological signs 3 weeks after cautery disbudding (17). The brain injury observed under gross examination may not have been severe enough to have been detected in the MR images in the present study. Our results highlight the variability and sensitivity within these techniques and justify the inclusion of multiple forms of assessment.

In conclusion, it appears that cautery disbudding regardless of iron application time can cause mild to moderate brain injury, with longer durations (e.g., 15 and 20 s) causing more severe and consistent brain injury. This pilot study is the first to relate iron application time to brain injury and based on our results together with the literature, short durations such as 5 s or less likely reduce the risk of brain injury of goat kids. However, future research should increase the number of animals used in this pilot study to better understand the effect of application time on brain injury. Investigations into the prevalence rates of edema for kids that experience iron applications of 5 s (or less) as well as the effect of NSAIDs on edema are required.

## Data Availability Statement

The raw data supporting the conclusions of this article will be made available by the authors, without undue reservation.

## Ethics Statement

The animal study was reviewed and approved by Institutional Animal Care and Use Committee at Iowa State University (Protocol number: IACUC-19-193). Written informed consent was obtained from the owners for the participation of their animals in this study.

## Author Contributions

Conceptualization: MH, JKS, TL, and PP. Methodology: MH, JKS, JF, TL, and PP. Formal analysis: MH. Investigation: MH, JF, JSS, JDS, TL, and PP. Writing – original draft preparation: MH. Writing – review and editing: MH, JKS, MS, JF, JSS, JDS, TL, and PP. Funding acquisition: PP, JKS, and MS. All authors contributed to the article and approved the submitted version.

## Conflict of Interest

The authors declare that the research was conducted in the absence of any commercial or financial relationships that could be construed as a potential conflict of interest.
