# Supplementary material for: Cautery Disbudding Iron Application Time and Brain Injury in Goat Kids: A Pilot Study
Source: Front Vet Sci. 2021 Jan 18;7:568750. doi: 10.3389/fvets.2020.568750 (PMC7849607; doi:10.3389/fvets.2020.568750)
Supplement: Supplementary file 1 [file Data_Sheet_1.docx]

**Supplementary Table 1.** Observations from the magnetic resonance (MR) images for goat kids that were cautery disbudded.

| **Kid** | **Horn bud (L/R)** | **Application time (s)** | **Grey matter edema (Y/N)** | **White matter edema (Y/N)** | **Shape** | **Size (cm)** | **GRE Void?** | **Comments** |
| --- | --- | --- | --- | --- | --- | --- | --- | --- |
| 1 | L | 5 | N | N | - | - | - | - |
|  | R | 20 | N | Y | Branching | 0.6 x 1.2 x 1.2 | N | Edema crossing multiple gyri |
| 2 | L | 15 | N | N | - | - | - | - |
|  | R | 10 | N | N | - | - | - | - |
| 3 | L | 10 | N | N | - | - | - | - |
|  | R | 5 | N | N | - | - | - | - |
| 4 | L | 20 | N | N | - | - | - | - |
|  | R | 15 | N | N | - | - | - | - |
| 5 | L | 5 | N | Y | Branching | 0.8 x 0.4 x 1.1 | N | Mainly one gyrus affected |
|  | R | 20 | N | Y | Branching | 2.3 x 0.6 x 0.9 | N | Edema crossing multiple gyri |
| 6 | L | 10 | N | N | - | - | - | - |
|  | R | 15 | N | Y | Branching | 0.5 x 1.4 x 0.2 | N | Edema crossing multiple gyri |

L: left side; R: right side; Y: yes; N: no; -: no evidence of injury

**Supplementary Table 2.** Summary of evidence for brain injury of goat kids that was evaluated by gross assessment, magnetic resonance (MR) imaging and histopathological changes associated with cautery iron application time (5, 10, 15, and 20 s).

|  |  |  | **Evidence of brain injury** | | |
| --- | --- | --- | --- | --- | --- |
| **Kid** | **Horn bud (L/R)** | **Application time (s)** | **Gross assessment (Y/N)** | **MR Imaging (Y/N)** | **Histopathological changes (Y/N)** |
| 1 | L | 5 | - | N | - |
|  | R | 20 | - | Y | - |
| 2 | L | 15 | - | N | - |
|  | R | 10 | - | N | - |
| 3 | L | 10 | N | N | - |
|  | R | 5 | N | N | - |
| 4 | L | 20 | Y | N | - |
|  | R | 15 | Y | N | - |
| 5 | L | 5 | N | Y | Y |
|  | R | 20 | Y | Y | Y |
| 6 | L | 10 | Y | N | Y |
|  | R | 15 | Y | Y | Y |

L: left; R: right; Y: yes; N: no; -: information was not collected for these kids.
